# Newly Developed Resorbable Magnesium Biomaterials for Orbital Floor Reconstruction in Caprine and Ovine Animal Models—A Prototype Design and Proof-of-Principle Study

**DOI:** 10.3390/jfb14070339

**Published:** 2023-06-27

**Authors:** Josip Tomic, Iris Wiederstein-Grasser, Monika Schanbacher, Annelie Martina Weinberg

**Affiliations:** 1 Department of Oral and Maxillofacial Surgery, Medical University of Graz, 8036 Graz, Austria; 2 Core Facility Experimental Biomodels, Division of Biomedical Research, Medical University of Graz, 8036 Graz, Austria; 3 Department of Orthopaedics and Trauma, Medical University of Graz, Auenbruggerplatz 5, 8036 Graz, Austria; anneliemartina.weinberg@medunigraz.at

**Keywords:** animal model, orbital surgery, biomaterial, trauma, proof of principle

## Abstract

Background: orbital floor fractures have not been reconstructed using magnesium biomaterials. Methods: To test technical feasibility, ex vivo caprine and ovine heads (n = 5) were used. Head tissues were harvested from pubescent animals (n = 5; mean age: 3.2 years; mean mass: 26.3 kg) and stored below 11 degrees for 7–10 days. All procedures were performed in a university animal resource facility. Two experienced maxillofacial surgeons performed orbital floor procedures in both orbits of all animals in a step-by-step preplanned dissection. A transconjunctival approach was chosen to repair the orbital floor with three different implants (i.e., magnesium implants; titanium mesh; and polydioxanone or PDO sheets). The position of each implant was evaluated by Cone-beam computed tomography (CBCT). Results: Axial, coronal, and sagittal plane images showed good positioning of the magnesium plates. The magnesium plates had a radiographic visibility similar to that of the PDO sheets but lower than that of the titanium mesh. Conclusions: The prototype design study showed a novel indication for magnesium biomaterials. Further testing of this new biomaterial may lead to the first resorbable biomaterial with good mechanical properties for extensive orbital wall defects.

## 1. Introduction

Orbital floor repair is a complex surgical procedure that requires specialized training and expertise to repair fractures or defects in the orbital floor [1]. The procedure can be performed as a standalone surgery or as a part of a larger surgical approach to address facial trauma, such as in the case of a car accident or sports injury [1]. A variety of materials have been used to reconstruct the orbital floor such as autogenous bone, alloplastic materials, and xenografts [2]. In the field of traumatology, there is an increasing demand for effective biomaterials that can enhance the patient’s recovery process [3,4,5]. Notably, magnesium-based biomaterials have not been used for orbital floor repair in humans but have gained immense attention because of their unique properties and potential for application in traumatology [6]. Magnesium materials have excellent mechanical properties and biocompatibility, making them ideal for use in fracture stabilization [3,4,5,7,8,9,10]. It is important to note that large orbital wall defects similar to orthopedic load-bearing fractures require material that has excellent hardness and good dimensional stability [10,11,12]. Furthermore, magnesium biomaterial has superior osseointegration properties, which have been shown to improve bone healing and regeneration [7,8,9]. In addition, magnesium biomaterials are biocompatible and resorbable, which means they do not cause any adverse reaction or immune responses and they can break down into harmless ions that can be excreted or absorbed by the body [13]. Of particular significance is that these biomaterials can be used in temporary implants where the implant will degrade once the bone has healed, making them ideal for use in pediatric traumatology [7,8,9]. Furthermore, the composition of magnesium biomaterials varies depending on the specific alloy composition. ZX00 magnesium material is a type of magnesium alloy that is composed of magnesium, zinc, and traces of other elements and is commonly used in traumatology due to its excellent corrosion resistance [14].

In contrast, resorbable or temporary polymer-based implants (i.e., PDO, polydioxanone) have been very popular to restore the structural integrity of the orbital floor [15,16]. Unfortunately, PDO foils have a slow rate of degradation, which may delay the healing process, and limited strength, which may limit their effectiveness in cases of significant trauma [2,15,16]. While small orbital wall defects can be treated with PDS foils, large orbital wall defects require strong and stable material to prevent herniation of the orbital contents [11,17]. Therefore, non-resorbable materials such as medical-grade titanium have been used for large defects, because they provide the necessary structural support [11,17]. While titanium mesh provides a long-lasting solution for large defects in the orbital wall, it may have a risk of infection, which can lead to complications and a longer recovery time [11,12]. While the risk of infection is low, it is not completely eliminated, and the infected titanium mesh may need to be removed [11,12]. However, removing the titanium mesh can be challenging, as it may have become integrated with the surrounding bone and soft tissues [11,12]. Therefore, new materials are required to capture the strength of both resorbable and non-resorbable materials.

A clear understanding of magnesium biomaterials and their potential use and visibility is critically important for developing application-specific implants. Another way to improve the effectiveness of orbital foils in osteosynthesis is to use new material compositions. Animal methods have long been used in research as they provide valuable insights into the feasibility and potential effectiveness of new interventions and devices [4,18,19]. It is important to note that animal models are readily available and safe, making them a convenient resource for researchers studying surgical techniques [4,18]. Therefore, the present ex vivo study aims to show the technical feasibility of utilizing a novel application of magnesium biomaterial in the development of medical implants. The results of this study can help to determine whether further development and testing of the device is warranted. Further testing of this new material may lead to the first resorbable biomaterial for small and large orbital wall defects.

## 2. Materials and Methods

### 2.1. Study Design

This prototype design study was conducted at the Core Facility of Experimental Biomodels, at the Division of Biomedical Research, and at the Department of Oral and Maxillofacial Surgery, at the Medical University of Graz, Austria. Animal models included sheep (n = 2) and goats (n = 3) weighing between 25 and 35 kilos for orbital floor reconstruction. No ethics approval was required. This study investigated the technical feasibility of newly developed magnesium orbital floor plates and tested preclinical application for the first time.

### 2.2. Three Implants

#### 2.2.1. Magnesium Implant

**Figure 1:**
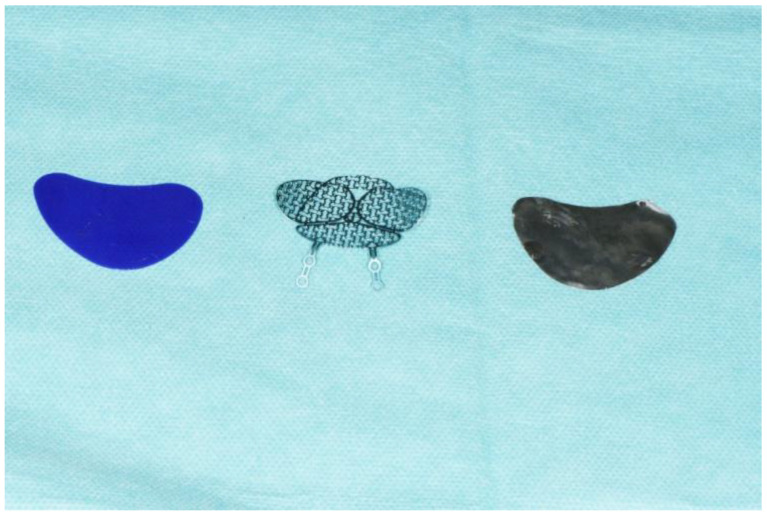
Implants used for orbital repair in this study: PDO, titanium mesh, Mg-based orbital floor plate (from left to right).

**Figure 2:**
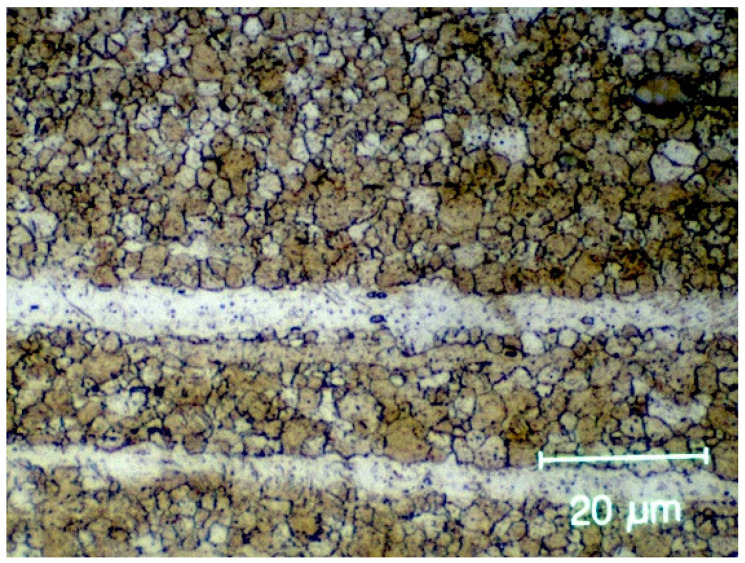
Orbital floor plates made from ZX00 implanted in sheep display an average grain size of 2.1 µm.

This study implant was composed of ZX00 magnesium (Mg-0.45Zn-0.45Ca) developed in close collaboration with the Laboratory of Metal Physics and Technology (LMPT) of the ETH Zürich, Switzerland. Mg-0.45Zn-0.45Ca alloy is a type of magnesium alloy that contains 0.45% of both zinc and calcium. It is a relatively new alloy that has been developed to improve the mechanical properties and corrosion resistance of magnesium. All magnesium implants had a size of 40 mm × 30 mm and a nominal thickness of 0.20 (Figure 1). The composition of Mg-0.45Zn-0.45Ca alloy (Figure 2):

- Magnesium (Mg): 98.1%.
- Zinc (Zn): 0.45%.
- Calcium (Ca): 0.45%.
- Other trace elements: <0.1%.

The addition of zinc and calcium to magnesium improved the alloy’s strength, corrosion resistance, and creep resistance. The alloy was also lightweight, making it ideal for applications that require a high strength-to-weight ratio. The extrusion process of Mg-0.45Zn-0.45Ca alloy required careful control of temperature and pressure to ensure that the resulting profile had the desired properties. The process was used to produce thin foils. The extrusion process of Mg-0.45Zn-0.45Ca alloy involved the following steps:

1. Billet preparation: the alloy was first cast into billets of appropriate size and shape.
2. Preheating: the billets were heated to a temperature of around 350–400 °C to make them soft and ductile.
3. Extrusion: The preheated billets were then loaded into an extrusion press and forced through a die of the desired shape and dimensions. The die was heated to prevent the alloy from sticking to it.
4. Cooling: the extruded profile was then cooled and straightened to the desired length.
5. Aging: the extruded profile was then aged at a temperature of around 175–200 °C for several hours to improve its strength and hardness.

#### 2.2.2. Titanium Mesh

Titanium mesh for orbital floor defects was made of medical-grade titanium along with small amounts of other metals, such as carbon, nitrogen, iron, and oxygen. Titanium is a biocompatible, corrosion-resistant, and flexible material. The mesh was a thin, flat sheet of titanium that was cut to the size and shape required to repair the orbital floor defect. The titanium mesh had a size of 39 × 30 mm and a plate thickness of 0.35 to 0.40 mm. The mesh was porous which allows for tissue ingrowth, which could help to anchor the implant and promote healing. Moreover, it was not coated with any biocompatible materials, such as hydroxyapatite or collagen.

#### 2.2.3. PDO

The composition of PDO foils used in this study was 100% polydioxanone (PDO). The PDO foils were thin and flexible biodegradable sheets. PDO foils can take several months to degrade completely. During surgery, the PDO foil was cut to the appropriate size and shape and placed over the damaged area of the orbital floor. The foil was then secured in place without using sutures or other surgical fixation methods. The PDO sheet (Ethicon, Germany) had a size of 40 mm × 30 mm and a nominal thickness of 0.25 mm. All implants are displayed in Figure 1.

### 2.3. Animal Models

In order to reduce the number of animals to a minimum, the aim was to carry out as many tests as possible per animal ex vivo. All animals were euthanized as part of other studies at the completion of live animal research being conducted under Austrian Federal Ministry of Education, Science and Research Committee approval, thus no live animals were used for this study. Animals were put into deep unconsciousness (anesthesia) and euthanized by an overdose of an intravenous injection of pentobarbital sodium (Exagon^®^), T61, or potassium umchloride (>2 mmol/kg i.v.). After disarticulation, ovine and caprine heads were stored below 11 degrees for 7–10 days. All head tissues were collected from pubescent animals (n = 5; mean age: 3.2 years; mean mass: 26.3 kg). Cadavers were abattoir specimens, and the institutional abattoir of Biomedical Research (BMF) of the Medical University of Graz, Austria was used.

**Figure 3:**
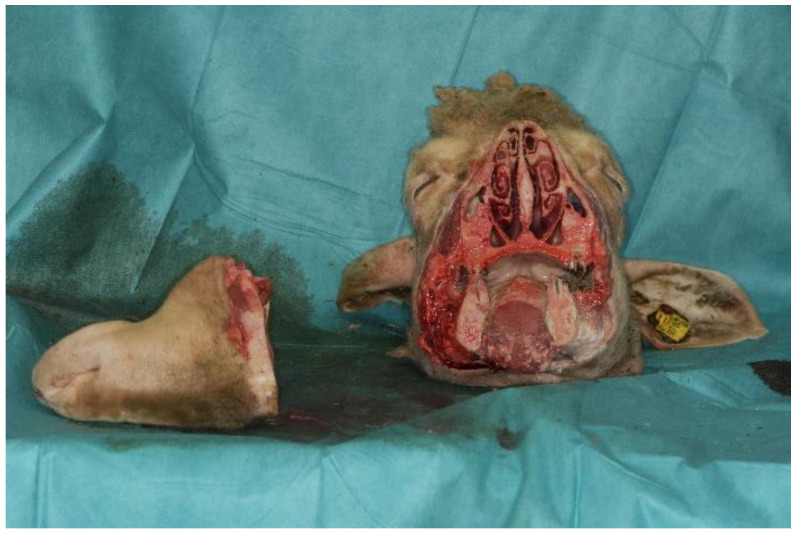
Adaptation of animal head with intact orbits in order to fit in the CBCT.

To fit in the CBCT, the snouts of the animals were sawed off (Figure 3), but orbits were left intact. After completion of the study, all animal tissues were disposed as biomedical waste.

### 2.4. Surgical Approach

**Figure 4:**
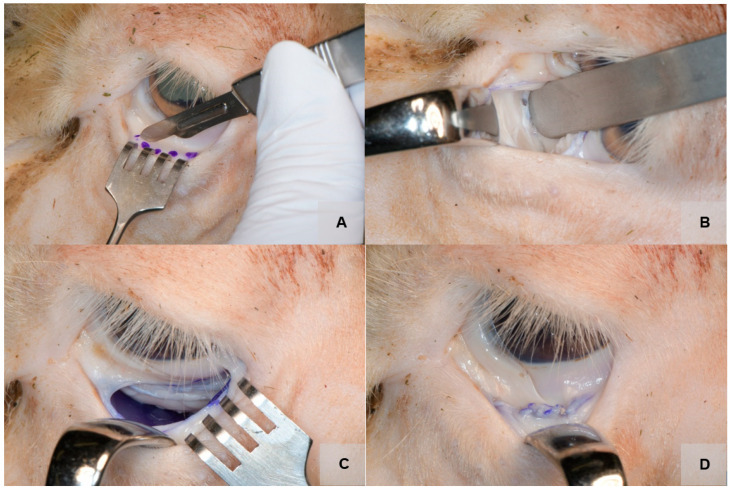
Orbital view: incision using a scalpel in the area of the marking (**A**); exposure of the orbital floor (**B**); insertion of the PDO sheet (**C**); wound closure with a resorbable suture (**D**).

Cadaver dissection techniques were used in order to carefully, systematically remove head tissues and prepare the orbits from deceased ovine and caprine bodies for research purposes. All surgical procedures took place in a controlled environment at the dissection room and were performed by two experienced surgeons. The same surgical protocol was followed. Before dissection began, the heads were cleaned and disinfected with the antiseptic povidone–iodine to reduce the risk of infection. The typical transconjunctival approach was chosen to access the orbital floor of the right and left orbits (see Figure 4) [20]. The dissection proceeded layer by layer, with each tissue identified. Then, the orbital septum was carefully dissected using scalpels, scissors, and forceps to avoid damaging the tissue or organs, and the infraorbital rim and the orbital floor were exposed. After blunt displacement of orbital fat, vessels, and nerves using blunt spatulas, the orbital floor was further exposed. A small fracture was then created by using a chisel. Ultimately, the implant was placed on the orbital floor (Figure 4), and the position and size of the implant was optically and radiologically checked. If the implant was too large, it was cut and adjusted to fit a smaller orbit. The orbital floor plate or implant was not fixed but inserted as tension-free as possible in the usual way after adaptation. In contrast, the titanium mesh (Orbital Plating System OPS 1.5, Medartis, Switzerland) was fixed with two 5 mm screws.

### 2.5. Study Outcomes

In the ex vivo examinations, parameters such as the position of the implant and radiologic visibility were investigated. Orbital floor fractures were managed with the use of a trial-specific application of the Mg-based implant. The primary objective of the trial was the suitability results of the magnesium biodegradable implant. Additionally, all three implants (magnesium orbital floor plates, titanium mesh, PDO sheets) were compared.

The hypothesis was defined as “Orbital floor repair is technically feasible with newly developed magnesium orbital floor plates”.

Technical feasibility was defined as:

1. Sufficient stability of the orbital plate against fracture and torsion during implantation.
2. Good position of the implant on the orbital floor.

## 3. Results

From March 2022 to July 2022, 15 orbital floor reconstructions (Figure 4) were performed on ex vivo ovine (n = 2) and caprine heads (n = 3). The characteristics of the procedure and the animal tissues are provided in Table 1. The operations performed in the study ranged in duration from 20 to 45 min. The caprine orbit and sheep orbit shared many similarities in terms of their anatomy. However, there were some notable differences between the two. One main difference was the size of the eye. Goats had a larger eye in proportion to their head size than sheep. Another difference was the shape of the orbit. The sheep orbit was more rounded, while the goat orbit was more oval-shaped. In addition, the shape of the animal orbital floor was a concave structure that formed the bottom of the orbit. The orbital floor had a curved shape with no sharp edges or protrusions and was relatively thin compared with other bones in the skull. The ovine and caprine orbital floor contained several foramina important for the passage of nerves and blood vessels that supply the eye and surrounding structures (e.g., the optic canal and infraorbital canal). Several bones, including the maxillary, palatine, zygomatic, frontal, and ethmoid bones, formed the orbital floor.

**Figure 5:**
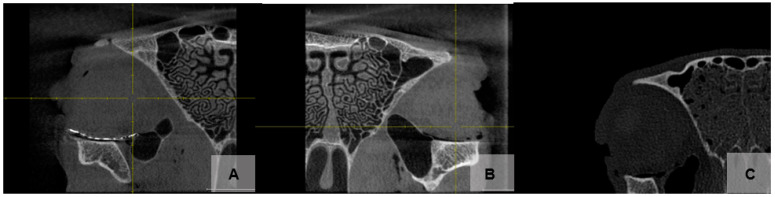
CBCT scans showing a 39 mm × 30 mm titanium mesh (right orbit, **A**), **PDO** (left orbit, **B**), and ZX00 magnesium implant (right orbit, **C**) on ovine orbital floor.

Two examiners assessed the position of the orbital implant using the CBCT scans and a rating scale of 0–10 [21]. All scans were performed using Planmeca ProMax 3D Max (Planmeca, Finland). The field of view was 15.0 × 6.9 cm, covering both orbits, with a 200 μm voxel size. An initial data set screening was performed on an MDNC-2221 monitor (resolution: 1600 × 1200; size: 432 × 324 mm; 59.9 Hz; Germany) using the Planmeca Romexis software (Planmeca, Finland). An evaluation of the reconstructed orbital floor by CBCT showed good positioning of all three tested implants (Figure 5). All of the materials were detectable in the CBCT scans. Visibility was significantly higher (*p* < 0.001) for titanium mesh than for PDO or ZX00 magnesium orbital floor plates. The animal bones were easily visible and distinguishable from surrounding soft tissues, as they had a similar Hounsfield value range as human bones, ranging from 700 to 3000 HU.

## 4. Discussion

The present study showed that orbital floor repair with Mg-based implants is technically feasible in caprine and ovine models. In addition, the inserted materials were visualized by CBCT scans. However, subjective visibility for different materials was lower for magnesium implants than titanium mesh implants. This could be explained by the different overall thickness of the plates (0.20 mm vs. 0.35/0.40).

The orbital floor is a common site of facial bone fractures, which are becoming more frequent due to an increasing number of accidents and violence [22]. As a result, small and large defects of the orbital floor require reconstruction with a multitude of different materials [11]. Authors have reported that biodegradable materials and titanium implants are good options for the surgical reconstruction of the orbital floor [21,23]. Despite their frequent use, the treatment of different orbital floor defects can be challenging due to the lower mechanical strength of polymer-based implants and the potential foreign body reactions and risk of infection of titanium-based materials [11,24,25]. In contrast, Mg-based implants have an elastic modulus similar to bone and good visibility on X-ray and fully degrade without residues [14,21].

Many studies have investigated polymer-based biomaterials for orbital floor repair [15,26,27,28]. Of note, one animal study investigated a polymer-based implant made of polytrimethylene carbonate (PTMC) [26]. Although polymeric materials are not osteoinductive, PTMC showed good osteoinduction [26]. Furthermore, another in vivo animal study reported that no implants were required, but cement composite was sufficient for the reconstruction of large orbital wall defects [29]. In general, polymer-based implants are the therapy of choice for the repair of small and titanium mesh for large orbital floor fractures [26,27,30].

Magnesium (Mg) is a biodegradable metal that has recently gained significant attention as a potential biomaterial for medical implants [31,32]. It is an abundant element in the human body and has excellent biocompatibility, which makes it an attractive option for use in medical implants [31,32]. Magnesium biomaterials have been shown to have promising mechanical and biological properties that make them suitable for various applications in orthopedics, cardiology, and dentistry [31,32]. The composition of magnesium biomaterials is critical in determining their mechanical and biological properties [5,33,34,35]. The elemental composition of magnesium biomaterials is typically at least 99% pure magnesium [5,33,34,35]. In addition to magnesium, some alloying elements can be added to improve the mechanical properties of the material [5,33,34,35]. The most commonly used alloying elements are aluminum, zinc, and manganese [35]. The addition of these elements can improve the strength, ductility, and corrosion resistance of the material [5,33,34,35]. The exact composition of magnesium biomaterials can vary depending on the specific application [35]. However, no negative effect has been reported on the regeneration of fractured human bone [18,36]. Additionally, the key principles of physiologic fracture healing include minimal physiologic movement of fixated fragments [37]. To improve biomechanical properties, magnesium alloys were combined with other elements, mainly rare earth elements or yttrium (W) [38]. As a consequence, high biocompatibility, high biofunctionality, and good osseointegration achieved with the use of magnesium alloys has greatly improved [5,33,34]. Similarly, a recent animal study on new magnesium membranes showed good performance and biodegradation for the treatment of bone defects according to the principles of guided bone regeneration (GBR) [5]. The biodegradation of magnesium implants is an important property resulting in fewer surgical interventions [5,18]. The degradation rate of magnesium material can range from a few weeks to several months, depending on the size and shape of the implant, the specific alloy used, and the processing techniques [5,18]. In addition, the biodegradation of magnesium implants depends on the pH value and the environment in which it is implanted [6]. Moreover, the degradation products of magnesium material include corrosion products [39]. These residuals can remain in the body for extended periods of time, but long-term exposure showed normal levels of serum magnesium and urine levels and no adverse effects on the liver and kidney function [39]. Furthermore, the surface treatment of magnesium implants has also played a role in improving biodegradation [6]. This coating of magnesium implants with PEO (plasma electrolytic oxidation) minimized hydrogen gas production and improved the mechanical stability of the implants [6]. Moreover, magnesium alloys have also been found to have antibacterial properties attributed to the release of magnesium ions, which can disrupt bacterial cell membranes and inhibit bacterial growth [40].

Several limitations of this study must be acknowledged, including the fact that the animal skulls used are different from the human skull. It is important to note that while there are some differences in the size and shape of the caprine and human orbital floors, both structures are essential for the proper functioning of the eye and protection of the surrounding tissues. The surface area of the ovine and caprine orbital floor was generally smaller than that of the human orbital floor. This is due to the fact that the overall size of their skulls is smaller than that of the human skull [41]. The orbital region of large animals has been validated in many studies [26,42]. It can be assumed that the weight of the orbital contents (eyeball, eyeball fat, and muscle) on the orbital implant was also similar [41]. From a mechanical standpoint, we can thus assume that an implant that works well in the sheep model will also work well in humans. However, it is known that a fibrous capsule develops after a fractured orbital floor, so from a biomechanical point of view it can be assumed that no major effects on the bony regeneration of orbital floors may be expected [26]. In summary, it is necessary to perform live animal experiments primarily to test the biocompatibility and biomechanics of these innovative magnesium implants for orbital reconstruction, prior to clinical testing.

## 5. Conclusions

This study showed that orbital floor repair is technically feasible with newly developed magnesium implants. The reconstruction of the orbital floor with this new material could provide an advantageous alternative to currently used biomaterials. Further testing of this new material could lead to the use of the first resorbable biomaterial with good mechanical properties for both small and large orbital wall defects.

## Figures and Tables

**Table 1:** Applicability of the ovine and caprine model for orbital floor reconstruction.

Procedure	Advantages	Disadvantages	Applicable for Simulation
Transconjunctival approach—orbital floor	Similar anatomy/tissue quality	Sparse orbital fat Large ovine nictitating membrane	Yes
Orbital floor reconstruction	Similar approach Partly absent orbital floor	Deficient eye muscle mass Differences in the shape of the orbit (rounded ovine vs. oval caprine orbit)	Yes

## Data Availability

The data presented in this study are available on request from the corresponding author. The data are not publicly available due its proprietary nature and confidentiality agreements.
